# Female Diseases from Stair-Climbing

**Published:** 1874-11

**Authors:** D. N. Kinsman


*DETROIT REVIEW OF MEDICINE—October*, 1874.

STAIR-CLIMBING—FEMALE DISEASES.—*By Prof. D. N. Kinsman.* "In answer to your question, 'Is stair-climbing a cause of disease among women, and why are they not as able to stand this kind of exercise as men?' I have no doubt that climbing stairs is productive of injury, but this is a result of other causes, and not of the simple fact of the ascent. Nature has provided a better support for the contents of the abdomen in women than men. The pelvis is more sloping, the ilia are broader and more capacious, the plain of the superior strait is such as to give a more oblique pressure of the superincumbent viscera, and the axis of the superior strait is directed backward, so the thrusting force is not so direct as in the case of males. Again, until puberty, the girl, in a rough and tumble fight, in a race, in everything, as far as physical force is concerned, is a match for her brother of the same age, unless she has been spoiled by her mother and made to believe it unladylike to romp and rough it. Then comes that mysterious change which the reproductive organs impress on her organism; all which is beantiful in outline and symmetry, and admirable in disposition; all which makes woman woman, and not man, is shaped by nature's plastic hand, and she stands the helpmeet for man. Now see what fashion does. The chest, which before has been free and unrestricted in motion, is encased in whalebone and steel; a pad goes here and a wad there, to produce the symmetrical ideal of this new comer—this novitiate—into life. The pelvis is expanded, the genital organs enlarged, her hips are made the point from which to suspend her clothing, pressure by the girdle extends across the upper part of the abdomen, limiting respiratory motion, and pressing the abdominal viscèra in the direction of least resistance. The uterus becomes congested; then result leucorrhœa, weakness of nerves, headache, nervousness, and the host of symptomatic disturbances we are taught to recognize as the evidences of a class of diseases now known as female diseases, and which have created the function of the gynæcologist. Woman cannot walk like a man, with the full swing of the arm, nor put her hand to her head when in full dress; and should a fly light on her nose, she could not get rid of it without poking it, or at it, with a fan or parasol. Thus pinioned, she tries to act out the promptings of nature, and fails. All this is the lot of the young woman; and middle life shows but few cases of sound womanhood. Now, under such circumstances, we can easily see why stair-climbing is an evil, and only evil continually among women. Climbing causes the heart to beat strongly, the lungs to expand fully, there is an impeded return of blood to the central organ, and the congestion of the pelvic organs is increased. There must be a chance for unloading this excess, and nature does it by an exhausting leucorrhœa, and, as a further sequel, we have this over-fullness of the uterus, and then all its train of consequences—flexions, versions and prolapsus. The muscular influences I do not discuss; they are patent to all."

